# Experimental *Bothrops atrox* envenomation: Efficacy of antivenom therapy and the combination of *Bothrops* antivenom with dexamethasone

**DOI:** 10.1371/journal.pntd.0005458

**Published:** 2017-03-17

**Authors:** Gabriella Neves Leal Santos Barreto, Sâmella Silva de Oliveira, Isabelle Valle dos Anjos, Hipocrates de Menezes Chalkidis, Rosa Helena Veras Mourão, Ana Maria Moura-da-Silva, Ida Sigueko Sano-Martins, Luis Roberto de Camargo Gonçalves

**Affiliations:** 1 Laboratorio de Fisiopatologia, Instituto Butantan, São Paulo, São Paulo, Brazil; 2 Laboratório de Pesquisas Zoológicas, Faculdades Integradas do Tapajós/Faculdade da Amazônia (FIT/UNAMA), Santarém, Pará, Brazil; 3 Laboratório de Bioprospecção e Biologia Experimental, Universidade Federal do Oeste do Pará, Santarém, Pará, Brazil; 4 Laboratorio de Imunopatologia, Instituto Butantan, São Paulo, São Paulo, Brazil; Instituto de Biomedicina de Valencia, SPAIN

## Abstract

*Bothrops atrox* snakes are the leading cause of snake bites in Northern Brazil. The venom of this snake is not included in the antigen pool used to obtain the *Bothrops* antivenom. There are discrepancies in reports on the effectiveness of this antivenom to treat victims bitten by *B*. *atrox* snakes. However, these studies were performed using a pre-incubation of the venom with the antivenom and, thus, did not simulate a true case of envenomation treatment. In addition, the local lesions induced by *Bothrops* venoms are not well resolved by antivenom therapy. Here, we investigated the efficacy of the *Bothrops* antivenom in treating the signs and symptoms caused by *B*. *atrox* venom in mice and evaluated whether the combination of dexamethasone and antivenom therapy enhanced the healing of local lesions induced by this envenomation. In animals that were administered the antivenom 10 minutes after the envenomation, we observed an important reduction of edema, dermonecrosis, and myonecrosis. When the antivenom was given 45 minutes after the envenomation, the edema and myonecrosis were reduced, and the fibrinogen levels and platelet counts were restored. The groups treated with the combination of antivenom and dexamethasone had an enhanced decrease in edema and a faster recovery of the damaged skeletal muscle. Our results show that *Bothrops* antivenom effectively treats the envenomation caused by *Bothrops atrox* and that the use of dexamethasone as an adjunct to the antivenom therapy could be useful to improve the treatment of local symptoms observed in envenomation caused by *Bothrops* snakes.

## Author summary

*Bothrops atrox* is the dominant species responsible for accidental human snake bites in Northern Brazil. The efficacy of antivenom therapy to correct the systemic disturbances, including hemostatic disorders, caused by Brazilian *Bothrops* is well known. However, two fundamental issues need to be addressed in this region. (1) There are concerns regarding the effectiveness of the antivenom to treat *Bothrops* snake bites in this region since *Bothrops atrox* venom is not used as an antigen to obtain the *Bothrops* antivenom in Brazil, and (2) the efficacy of the antivenom therapy in reversing local injuries induced by *Bothrops* venoms is low. Thus, our study aimed to assess the effectiveness of antivenom therapy alone or in combination with dexamethasone to treat experimental envenomation induced by *Bothrops atrox* venom in mice. Our results showed that the Brazilian *Bothrops* antivenom effectively reversed the systemic disturbances caused by this envenomation and combining the antivenom therapy with dexamethasone accelerated the regression of inflammatory edema and the regeneration of skeletal muscle that was damaged by the venom.

## Introduction

Snake bites are a public health problem. They are associated with poverty and occupation and affect the population of rural areas [1,2]. Despite no longer being listed on the World Health Organization list of neglected tropical diseases, snake bites continue to affect thousands of victims every year [3,4].

In Brazil, approximately 26,000 cases occur per year, of which an average of 0.39% cause death and 1.72% heal with sequelae [4,5]. Northern Brazil is the region with the highest incidence of cases/100,000 inhabitants, and the state of Pará has the second-highest rate in the country, after Roraima state [5].

*Bothrops atrox* snakes are distributed through the South American Amazon region [6,7]. In Northern Brazil, this species causes the majority of bites, but its venom is not used as an antigen in the production of the *Bothrops* antivenom. The antivenom produced in Brazil is obtained using a pool of antigens containing the following venoms: 50% *Bothrops jararaca*, 12.5% *B*. *jararacussu*, 12.5% *B*. *alternatus*, 12.5% *B*. *moojeni*, and 12.5% *B*. *neuwiedi*. This antigen formulation was achieved studying the cross-reactivity of monovalent antivenoms to venoms of ten *Bothrops* species [8].

Some studies have suggested that the *Bothrops* antivenom produced in Brazil does not effectively neutralize the *B*. *atrox* venom [9,10], but other studies have shown that the *Bothrops* antivenom can neutralize the toxins of this venom [11–14]. All these studies were performed using a prior incubation of the venom with the antivenom, which does not represent the actual situation where the treatment with antivenom follows the envenomation after the onset of the primary venom-induced effects. A clinical study also showed the efficacy of the *Bothrops* antivenom in the treatment of *Bothrops atrox* snakebites [15].

Similar to other *Bothrops* venoms, *B*. *atrox* bites cause hemostatic disturbances and local reactions including edema, hemorrhage, and necrosis, which can progress to tissue losses that lead to sequelae [15,16].

The specific antivenom therapy is the only recommended procedure for treating envenomation by snake bites [17,18]. Neutralization of the toxins by the antivenom results in an efficient restoration of the coagulation factors but does not reverse the locally established lesions or the inflammatory mediators released by the damaged tissue [19–21].

Studies have shown that eicosanoids are the major mediators of the inflammatory edema induced by *Bothrops* venoms [22–27]. One study showed that the use of a combination of dexamethasone with antivenom reverses the *Bothrops jararaca* venom-induced edema when administered after venom injection in mice. However, the individual treatments with antivenom or with dexamethasone did not decrease the edema [27]. This combined therapy was also effective in reducing the myotoxic effect induced by the venoms of *B*. *jararaca* and *B*. *jararacussu* [28].

In this study, we tested the efficacy of *Bothrops* antivenom to treat the signs and symptoms of experimental *B*. *atrox* venom-induced toxic effects in mice. We also assessed whether combining the antivenom with dexamethasone would be beneficial to treat the pathophysiological effects of this envenomation.

Our results show that the Brazilian *Bothrops* antivenom effectively reverses the coagulopathy induced in mice by *Bothrops atrox* venom. Furthermore, the combination of dexamethasone with the antivenom improved the reversal of the inflammatory edema and the regeneration of the skeletal muscle that was damaged by the venom.

## Materials and methods

### Ethics statements

All the experimental protocols used in this work were reviewed and approved by Institute Butantan Animal Care and Use Committee (protocol n° 1249/14). These procedures are in accordance with standards outlined by Brazilian laws for use of experimental animals, and with ethical principles adopted by the Brazilian College of Animal Experimentation.

### Venom, antivenom and dexamethasone

Venom of wild *Bothrops atrox* (BaV) was obtained from 22 snakes of both sexes, collected in Santarém, Pará, Brazil (SISBio license 32098–1). Each venom sample was extracted individually and freeze-dried, after which the samples were pooled in equal proportions, and stored at –20°C until use. *Bothrops* antivenom produced by Instituto Butantan (batch 0609/68/C) and injectable dexamethasone (Aché, Brazil) were used. One mL of *Bothrops* antivenom neutralizes 5 mg of *Bothrops jararaca* venom.

### Animals

Male Swiss mice (18–22 g body weight) were obtained from the animal housing facility of the Instituto Butantan. The animals were maintained in a 12:12 hour light:dark cycle and received water and food ad libitum.

### Toxic activities

Before evaluating the effectiveness of treatments in local manifestations of *Bothrops atrox* envenomation, the minimum doses of BaV required to induce the pathophysiological activities studied (edema, hemorrhage, dermonecrosis and muscle damage) were determined. The details of the methodologies for assessing these toxic activities of the BaV were previously described [12,29].

### Treatment protocols

After determining the minimum doses required to induce the pathophysiological manifestations to be studied, the challenge doses were established for use in the protocols for treatments to be administered 10 or 45 minutes after the venom injection. The treatment groups consisted of (1) antivenom (AV) administered intravenously (0.2 mL/animal); (2) dexamethasone (Dexa) administered intraperitoneally (1 mg/kg); (3) the combined administration of AV and Dexa protocols; and (4) control group that was untreated (5 mice/group). The doses of antivenom and Dexa were those used in a previous study [27].

### Edematogenic activity

Edema was induced by injecting 0.75 μg of BaV in 30 μL of sterile saline into one hind foot pad of each mouse, and the same volume of saline was injected into the contralateral paw. The volume of both paws was verified by plethysmometry 1, 2, 4, 6 and 24 hours after the venom injection. The edematogenic activity results were expressed as the percent difference between the venom- and saline-injected paws.

### Hemorrhagic activity

Hemorrhage was induced by injecting 3.3 μg of BAV in 100 μL of sterile saline intradermally in the ventral region of the abdomen. Two hours after the injection, mice were euthanized, their skins were removed and the diameter of the hemorrhagic halo on the inner side of the skin was determined for each group.

### Dermonecrotic activity

The dermonecrosis diameter was obtained in a similar protocol. The mice were injected intradermally in the ventral region with 30 μg of BAV in 100 μL of sterile saline. At 48 hours after injection, the mice were euthanized, their skins were removed and the diameter of the dermonecrotic halo on the inner side of the skin was determined for each group [29].

### Myotoxic activity

Myonecrosis was evaluated by quantifying the plasma creatine kinase (CK) activity according to the instructions for the CK-NAC Liquiform (LabTest, Brazil). Each mouse received an i.m. injection of 40 μg of BaV in 40 μL of saline into the gastrocnemius muscle. At 3 and 24 h after the venom injection, blood samples were collected by an orbital puncture for determination of the serum CK levels, which were expressed as units/L.

### Muscle regeneration

Muscle regeneration was evaluated by the quantification of the muscle tissue creatine kinase (residual CK) activity and by histological analysis. Each mouse received an i.m. injection of 40 μg of BaV in 40 μL of saline into the right gastrocnemius muscle and the same volume of saline into the left gastrocnemius muscle followed by the treatment protocols. Groups of mice were euthanized 1, 4, 7, 14, 21 and 28 days after the venom injection, and both gastrocnemius muscles were collected, weighed, and homogenized in 4 mL phosphate buffered saline (pH 7.4) containing 0.1% Triton X-100. After the centrifugation (2100 *g*/5 minutes), the supernatant was collected for determination of the residual CK (CK-NAC Liquiform, LabTest). The results are expressed as the percentage of CK obtained in the venom-muscle injected (residual CK) compared with the CK content of the control contralateral muscle.

In another set of experiments, the injected muscles were collected for histological analysis. They were fixed in Bouin’s solution, embedded in paraffin, sectioned and stained with hematoxylin and eosin (HE) for light microscopic analysis.

### Fibrinogen levels and platelet counts

Four groups of mice were envenomated as described for the analysis of the myotoxic activity (40 μg/40 μL, i.m.) and treated 45 minutes later. Six hours after the envenomation, blood was collected (9:1, v:v) in 13 mmol sodium citrate with 2% of *Bothrops* antivenom to neutralize venom in the sample [19]. The fibrinogen concentration was evaluated in citrated plasma [30] and the platelet count was determined in EDTA-anticoagulated blood using an automated cell counter (Mindray BC-2800 Vet, Nanshan, Shenzhen, China).

### Statistical analysis

One-way ANOVA followed by the Tukey test was used to evaluate significant differences between the treated and non-treated groups. The data are expressed as the means ± SEM, and differences were considered statistically significant when the p values were < 0.05.

## Results

### Local effects

#### Edema

**Fig 1:**
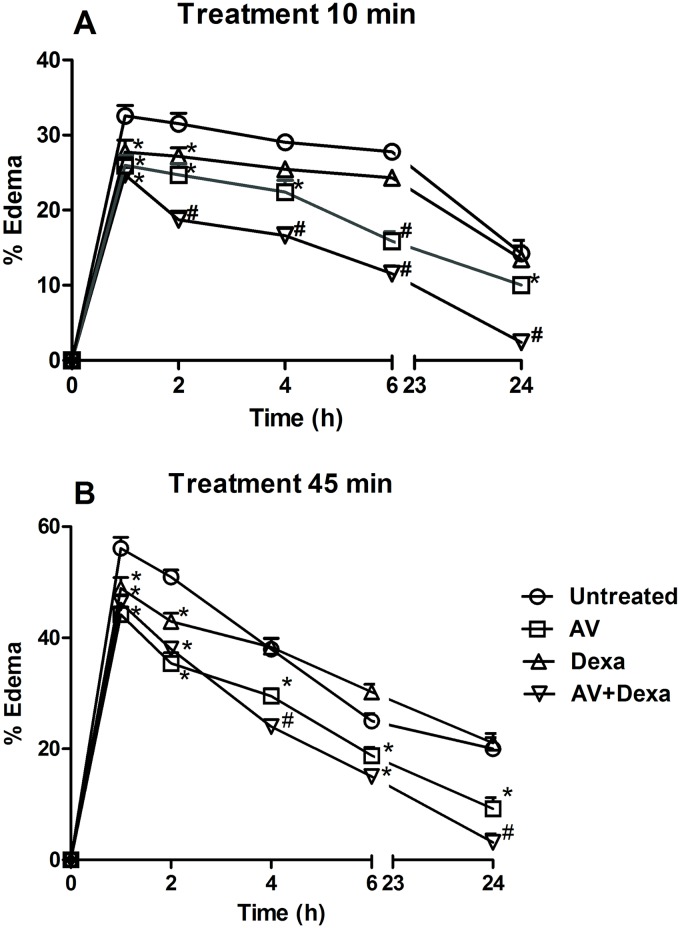
Time-course of *B*. *atrox* venom-induced edema. Groups (n = 5/group) were treated with AV (0.2 mL, i. v.), Dexa (1 mg/kg, i. p.) or AV + Dexa by the same routes and doses 10 (A), or 45 minutes (B) after injection of 0.75 μg of *B*. *atrox* venom into the footpad, and the results were compared to those obtained in the untreated control group. The edema (%) is expressed as the means ± S.E. Statistically significant (p < 0.05) differences compared with the control group (*) or with all other groups (#) are indicated.

The venom of *Bothrops atrox* induced an inflammatory edema with a peak in the first hour after the venom injection followed by progressive decreases. However, edema was still present after 24 h (Fig 1A and 1B). When administered ten minutes after the envenomation, all treatments significantly reduced the edema until the 2nd hour. From the fourth to 24th hours, only the groups treated with antivenom or antivenom + dexamethasone demonstrated a reduction of the edema compared with the untreated control group. Among these, the group treated with antivenom + dexamethasone demonstrated less edema than that observed in the group treated with antivenom alone (Fig 1A).

In the groups treated 45 minutes after the envenomation, the mice treated with antivenom or with antivenom + dexamethasone demonstrated significantly less edema than the untreated control group at all the times evaluated. For this set of treatments, the group treated with antivenom + dexamethasone also demonstrated less edema than that observed in the antivenom-treated group (Fig 1B).

#### Hemorrhage and dermonecrosis

**Fig 2:**
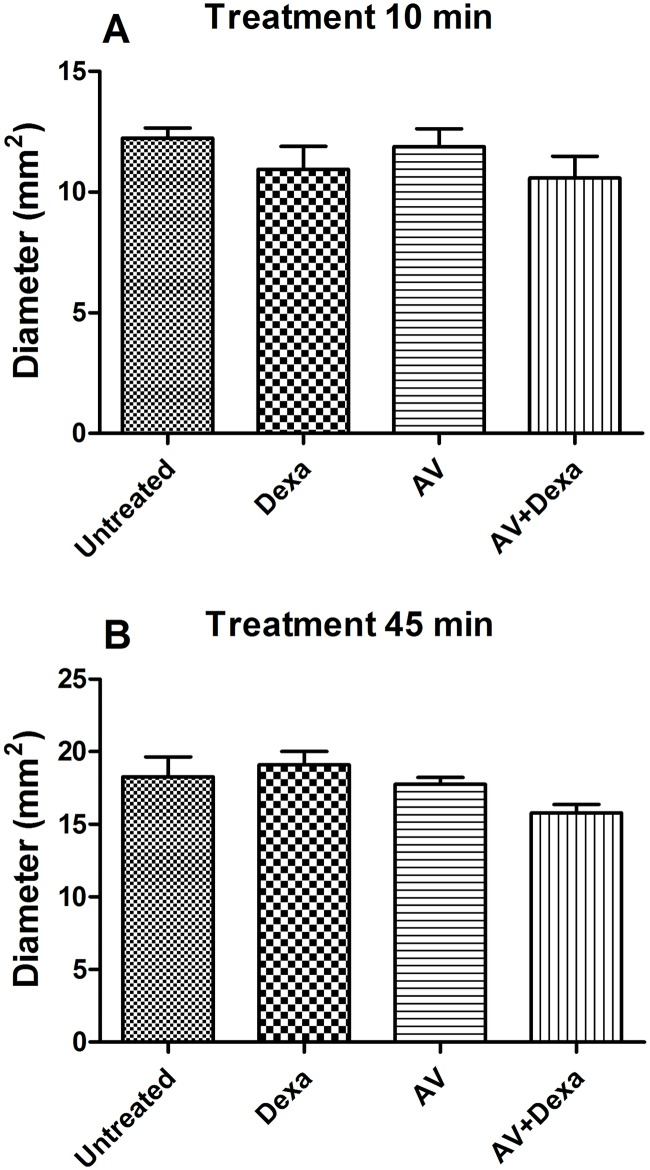
Local hemorrhage induced by *B*. *atrox* venom. Groups of mice (n = 5/group) were treated with AV (0.2 mL, i. v.), Dexa (1 mg/kg, i. p.) or AV + Dexa by the same routes and doses 10 (A), or 45 minutes (B) after i.d. injection of 3.3 μg of *B*. *atrox* venom, and the results were compared to the untreated control group. The degree of hemorrhage is expressed as the means ± S.E of the diameters of the spots on the inner surface of the injected skin 2 h after the venom injection.

**Fig 3:**
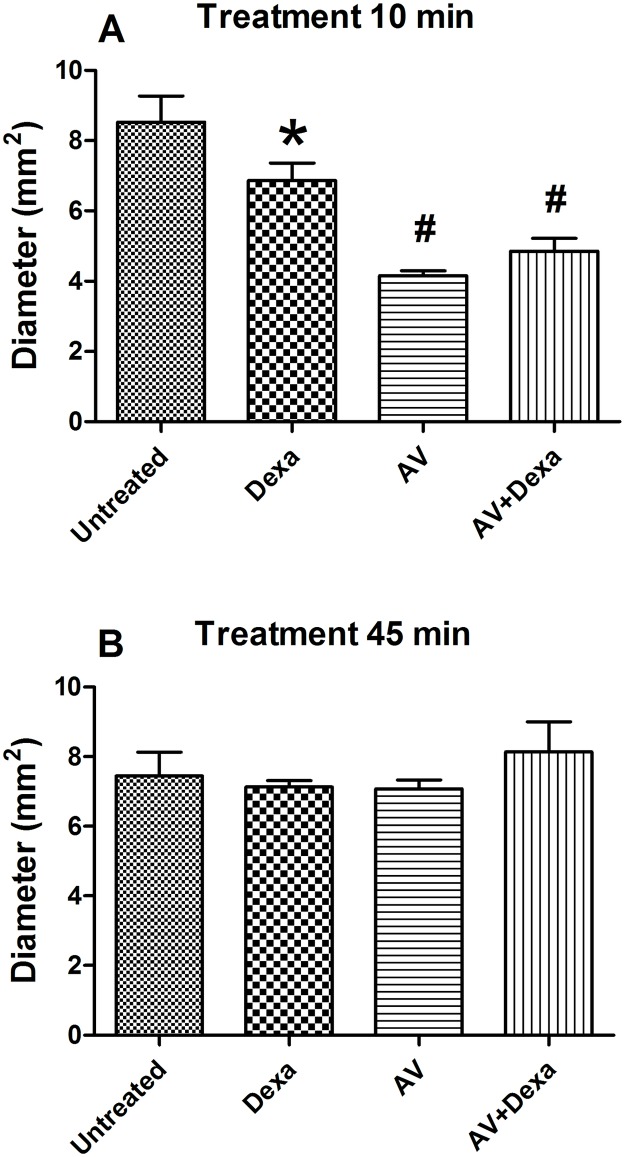
Dermonecrosis induced by *B*. *atrox* venom. Groups of mice (n = 5/group) were treated with AV (0.2 mL, i. v.), Dexa (1 mg/kg, i. p.) or AV + Dexa by the same routes and doses 10 (A), or 45 minutes (B) after i.d. injection of 30 μg of *B*. *atrox* venom, and the results were compared to the untreated control group. The dermonecrosis was expressed as the means ± S.E. of the diameters of the spots on the inner surface of the injected skin 48 h after the venom injection. The statistically significant (p < 0.05) differences from the untreated control group (#) or from all other groups (*) are indicated.

None of the treatments reduced the hemorrhage, even when antivenom and/or dexamethasone were administered as early as 10 minutes after intradermal injection of the venom (Fig 2A and 2B). However, all treatments resulted in a significant reduction in the necrotic area when administered 10 minutes after envenomation. The groups treated with antivenom or antivenom + dexamethasone demonstrated smaller necrotic areas than those observed in the group treated with dexamethasone alone (Fig 3A). In contrast, treatments administered 45 minutes after the intradermal injections of the venom were not effective (Fig 3B).

#### Myonecrosis

**Fig 4:**
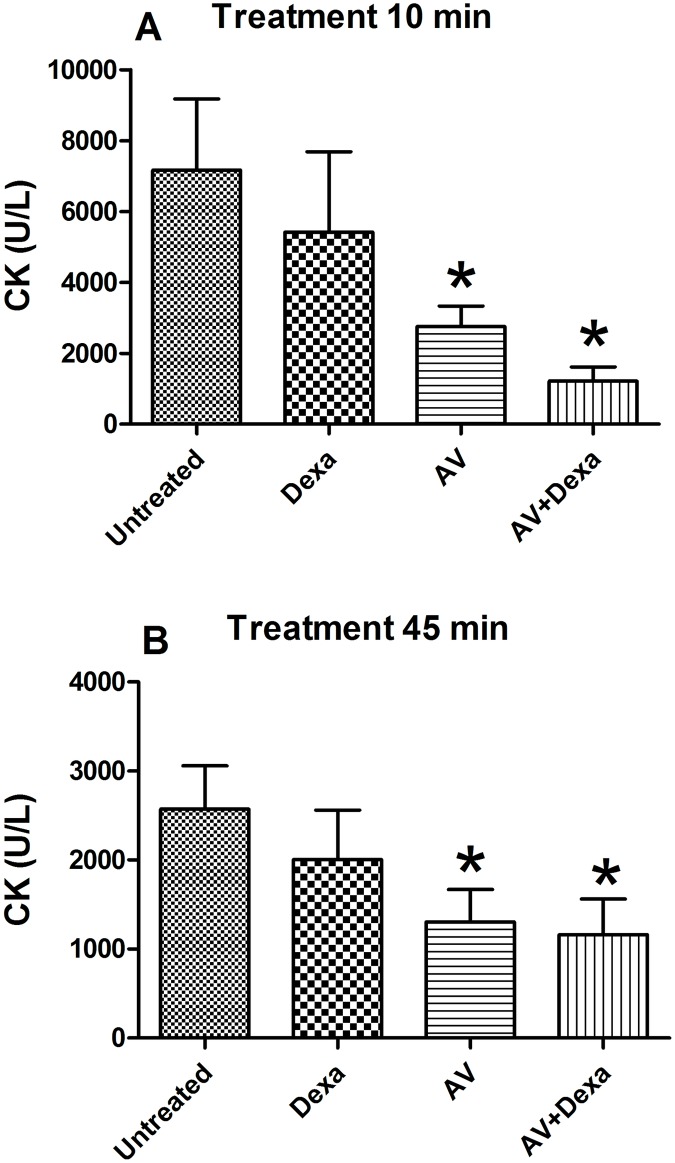
Myonecrosis induced by *B*. *atrox* venom. The muscle damage was evaluated by measuring the levels of CK in serum, 3 h after the i.m. injection of *B*. *atrox* venom (40 μg) into the right calf muscles of the mice. Groups (n = 5/group) were treated with AV (0.2 mL, i. v.), Dexa (1 mg/kg, i. p.) or AV + Dexa by the same routes and doses 10 (A), or 45 minutes (B) after the envenomation. The results are expressed as the means ± S.E., and the statistically significant (p<0.05) differences from the untreated control group are indicated (*).

The animals that were treated with antivenom or with antivenom + dexamethasone 10 or 45 minutes after the intramuscular injection of the venom demonstrated creatine kinase levels (3 h) that were significantly lower than those observed in the untreated group. However, there was no statistically significant reduction of this parameter in the groups that were treated with dexamethasone alone compared to the serum CK levels observed in the untreated group (Fig 4A and 4B).

#### Muscle regeneration

**Fig 5:**
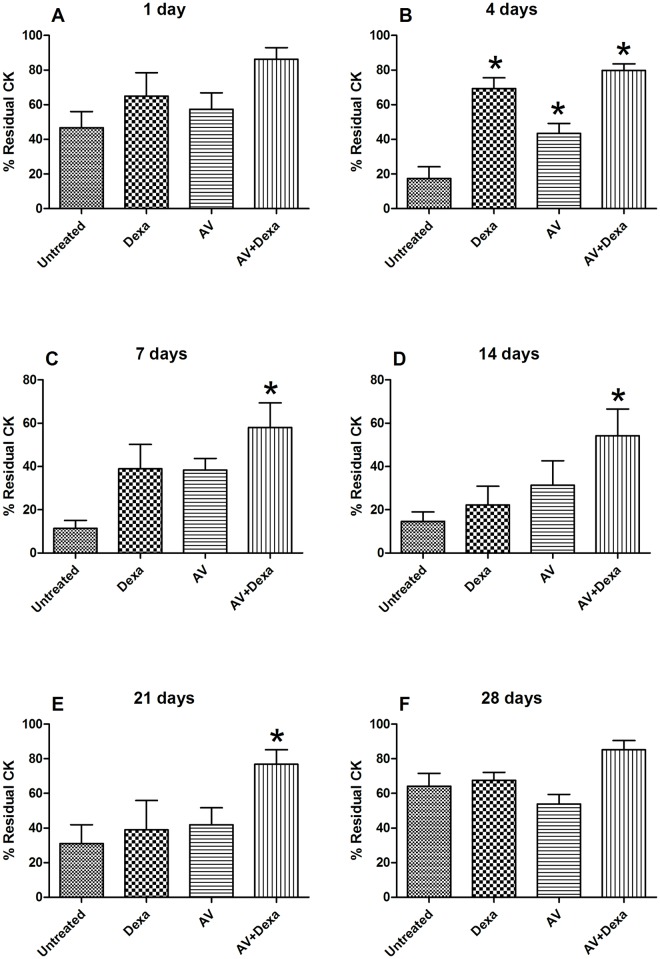
Kinetics of muscle injury and regeneration after envenomation by *B*. *atrox*. At 1 (A), 4 (B), 7 (C), 14 (D), 21 (E), or 28 (F) days after the i.m. injection of *B*. *atrox* venom (40 μg), the CK contents were evaluated in the envenomated and the contralateral saline-injected calf muscles. Groups (n = 5/group) were treated with AV (0.2 mL, i. v.), Dexa (1 mg/kg, i. p.) or AV + Dexa by the same routes and doses 45 minutes after the envenomation. The results are expressed as the means ± S.E. and presented as the percentage of the CK content compared to the saline-injected muscle. The statistically significant (p < 0.05) differences from the untreated control group are indicated (*).

The regeneration of the muscle tissue after the envenomation was measured as the residual content of creatine kinase in the envenomated muscle compared to the contralateral muscle. The results showed that there were no significant differences among the groups 1 day after the envenomation (Fig 5A). In contrast, four days after the envenomation, the groups treated with antivenom, with antivenom + dexamethasone or with dexamethasone demonstrated a residual creatine kinase significantly higher than that observed in the untreated group (Fig 5B).

From the 7th to the 21st day after the envenomation, only the group treated with antivenom + dexamethasone demonstrated significantly higher levels of creatine kinase (Fig 5C, 5D and 5F). At the 28th day after the envenomation, levels of residual creatine kinase were similar in all groups (Fig 5G).

**Fig 6:**
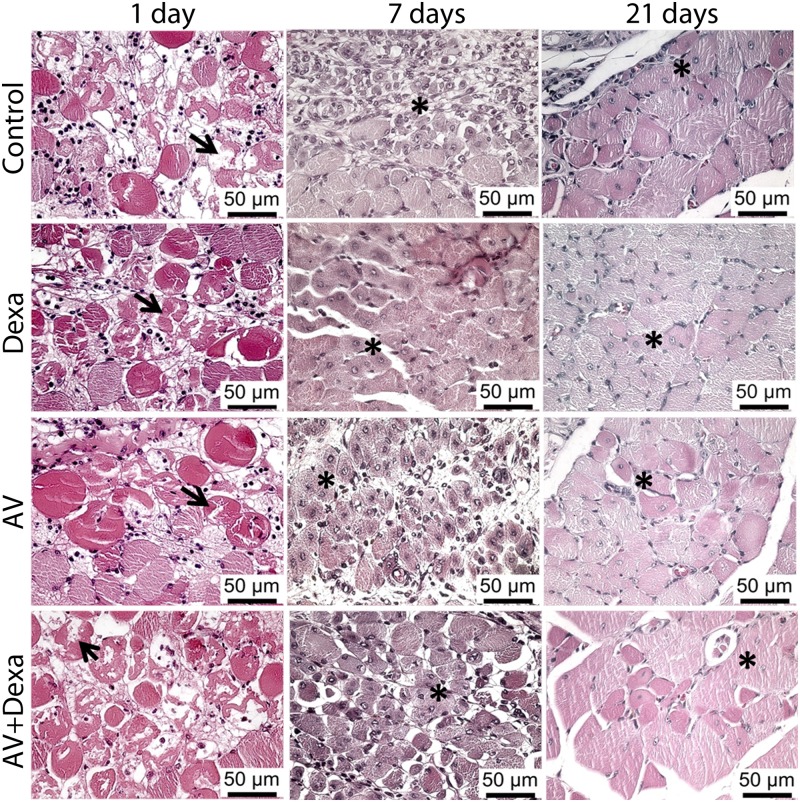
Morphology of the skeletal muscle injury, and regeneration after envenomation by *B*. *atrox*. Groups were envenomated by an i.m. injection of 40 μg (40 μL) of *B*. *atrox* venom and treated 45 minutes later with AV (0.2 mL, i.v.), Dexa (1.0 mg/kg, i.p.), or AV + Dexa by the same route and doses. The morphology of the treated groups was compared to that of the untreated control group. The calf muscles of the animals were collected 1, 7 or 21 days after the envenomation and processed as described in Materials and Methods. At 1 day, necrotic areas were observed in all groups (arrows). Seven days after envenomation, some areas of regenerated skeletal muscle cells with central nuclei were observed in all groups (*). At 21 days post-envenomation, regenerated muscle fibers with centrally located nuclei were observed in all groups (*). In all cases, paraffin sections were stained with hematoxylin and eosin.

The histologic analysis of the muscle regenerative process showed necrotic areas on the first day after the intramuscular venom injection in all groups. On the 7th day after the envenomation, we observed areas of inflammatory infiltration in the untreated control groups and those treated only with antivenom, and areas of regeneration were noted in all groups. On the 21st day after envenomation, the regenerative process, which is characterized by the presence of myocytes with a central nucleus, was more evident in all groups, but we did not observe any morphological differences among the studied groups (Fig 6).

### Systemic effects

To evaluate the effects of the treatments on the systemic disturbances induced by *B*. *atrox* venom, we assayed the plasma fibrinogen levels and the numbers of platelets.

**Fig 7:**
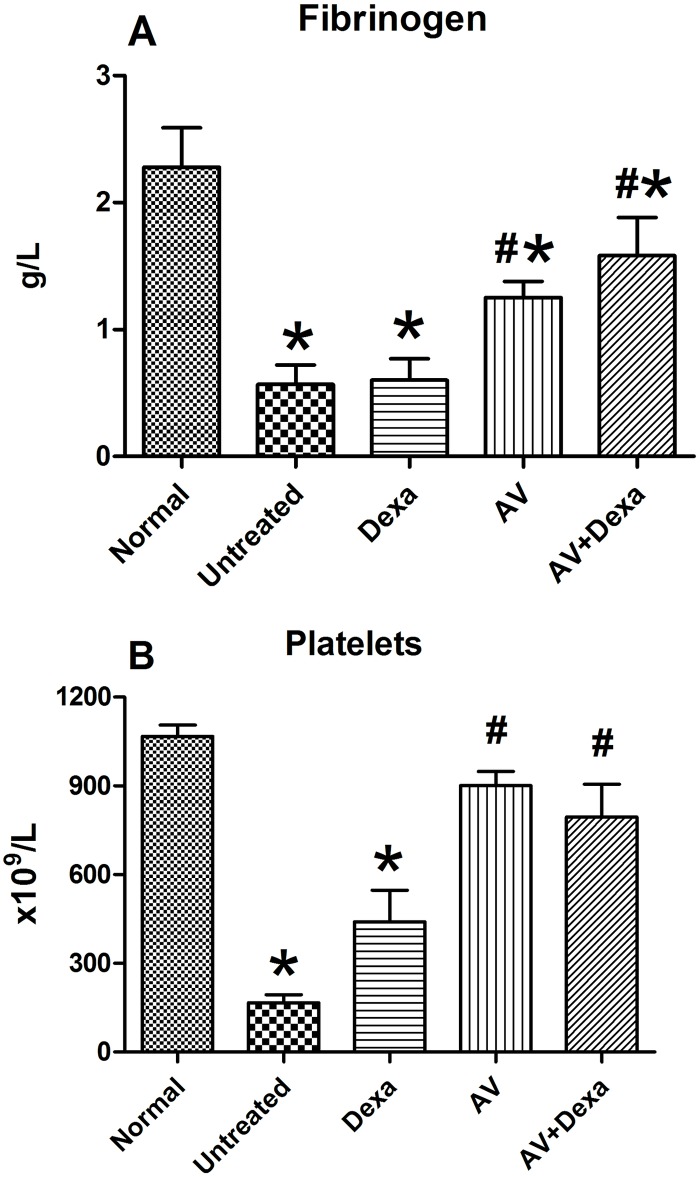
Fibrinogen levels (A) and platelet counts (B) in mice envenomated with *B*. *atrox* venom. Animals (n = 4/group) were injected (i.m.) with *B*. *atrox* venom (40 μg/40 μL). Forty-five minutes later, groups were treated with AV (0.2 mL, i.v.), Dexa (1.0 mg/kg), AV + Dexa, or remained untreated, and blood was collected 6 h after the envenomation. The platelets were counted in the whole blood, and fibrinogen was evaluated in citrated plasma as described in Materials and Methods. The results are expressed as the means ± S.E. and the statistically significant (p<0.05) differences from the non-envenomated animals (*) and the untreated group (#) are indicated

Our results showed that there was significant fibrinogen consumption in the untreated animals and those treated with dexamethasone. Six hours after the treatments with antivenom or antivenom + dexamethasone, the fibrinogen levels increased to reach hemostatic levels, and in both cases, the values were significantly higher than those of the untreated animals and those treated only with dexamethasone (Fig 7A).

Severe thrombocytopenia was observed in the untreated or dexamethasone-treated groups. Similar to the results for fibrinogen consumption, the numbers of platelets in the groups of mice treated with antivenom or antivenom + dexamethasone were restored (Fig 7B).

## Discussion

Our results showed that the *Bothrops* antivenom can counteract the systemic and local signs and symptoms of *Bothrops atrox* envenomation in mice.

*Bothrops atrox* is the species of snake that causes the most bites in humans in Northern Brazil and Amazon region. However, *B*. *atrox* venom is not included in the pool of antigens that is used to obtain the antivenom. Some *in vitro* studies have suggested that the *Bothrops* antivenom does not effectively neutralize the *Bothrops atrox* venom [9,10]. In contrast, pre-clinical, antivenomic, and clinical studies have shown that the *Bothrops* antivenom effectively neutralizes *B*. *atrox* venom [12–15]. However, with the exceptions of the clinical [15] and the antivenomic [13] studies, all other studies of *Bothrops* venom have evaluated its biological and enzymatic activities, including the lethal, hemorrhagic, coagulant, defibrinating, and myotoxic effects, after preincubating the venom with the antivenom.

In the present study, we used a model that mimics treatment of a snake envenomation, and our data corroborate those studies that showed the efficacy of the antivenom. Thus, the groups treated with the antivenom demonstrated a significant reduction of all the effects studied except for the local hemorrhage. The efficacy of the antivenom against the local symptoms was more obvious when it was applied soon after the experimental envenomation, as has also been observed in the clinical studies [19]. In the case of the edema, the antivenom was effective even when applied 45 minutes after the injection of the *B*. *atrox* venom into the footpads of mice. It is interesting to note that following envenomation with *B*. *jararaca* venom, the main antigen used to obtain the *Bothrops* antivenom, the edema was not affected when the antivenom was applied 45 minutes after the experimental envenomation [27].

All the animals injected with *B*. *atrox* venom demonstrated signs and symptoms of the envenomation, such as edema, local hemorrhage, fibrinogen consumption, and thrombocytopenia, at the times that the treatments were applied. Once the antivenom had neutralized the venom, six hours after the envenomation, the fibrinogen levels and the platelet numbers were within hemostatic levels, although lower than the levels in the non-envenomed group. Clinicians use the restoration of the hemostatic parameters as indicators of the efficacy of the antivenom therapy [31]. However, the inflammatory process and the healing of established lesions have longer time-courses and involve the participation of some endogenous mediators that are not influenced by the antivenom [21].

Eicosanoids are main mediators of inflammatory response induced by *Bothrops* venoms [22–27]. From this point of view, the use of anti-inflammatory drugs in combination with the antivenom therapy could reduce the time for the tissue regeneration and potentially improve the functions of the healed tissue. With this rationale, the use of dexamethasone combined to *Bothrops* antivenom presented a better result than specific inhibitors of cyclooxygenase or lipoxygenase pathways, such as indomethacin or NDGA, in reversing the inflammatory edema induced by the *Bothrops jararaca* venom into the paw of mice [27].

Edema is the most common inflammatory sign of envenomation by *Bothrops* snake bites in humans. It can affect the entire bitten limb and can evolve to a compartmental syndrome that increases the risk of permanent sequelae [20]. For the edema induced by *B*. *atrox*, the antivenom + dexamethasone combination was the most efficient treatment when applied 10 or 45 minutes after the envenomation. Nevertheless, in contrast to the results obtained using *B*. *jararaca* venom, the present results showed that this therapeutic combination caused a significant reduction of the edema in the second hour and subsequently after envenomation [27]. These data suggest that in addition to eicosanoids, other endogenous mediators such as histamine may participate in the snake venom-induced inflammatory edema [32].

Having confirmed the efficacy of this treatment for edema, we evaluated whether it could affect other toxic effects induced by this venom such as local hemorrhage, dermonecrosis, and myonecrosis. We also determined whether this therapeutic strategy could have an impact on the regeneration of damaged muscle and some hemostatic parameters.

The local hemorrhage induced by viperid venoms is one of the most rapid pathological symptoms that occurs after contact of the venom with the connective tissue [24,33,34]. This effect is induced by Snake Venom Metalloproteinases (SVMPs) that are present in these venoms [35]. In the venom of the *B*. *atrox*, SVMPs are the most abundant toxins, representing more than 50% of the crude venom [13,14,36]. These early effect of the venom could explain the lack of efficacy of an antivenom, even when applied 10 minutes after venom injection, despite the fact that the antivenom has antibodies that can neutralize these SVMPs when incubated with the venom [12]. Treatment with dexamethasone, alone or combined with the antivenom, also did not affect the hemorrhagic lesion induced by this venom. This result is consistent with the fact that eicosanoids do not participate in the pharmacological mechanisms of the local hemorrhage induced by *Bothrops jararaca* venom [24].

The SVMPs also participate strongly in the dermonecrosis by acting directly on the components of the connective tissue [37]. The SVMPs induce their effects by generating cytokines, such as TNF-α, through the cleavage of pro-TNF-α [38,39]. Nevertheless, our results have shown that all the treatments were effective in reducing the necrotic area of the injected skin when applied 10 minutes after the venom injection, and none of them were effective when applied 45 after the experimental envenomation.

These data indicated that the antivenom could neutralize the effects of the venom when administered early after the envenomation, which suggested that the onset of the necrotic lesions occurs later than that of the hemorrhagic lesions and that neutralization of SVMPs by antivenoms could prevent the development of necrosis. Furthermore, the group treated with dexamethasone demonstrated a significant reduction of the necrosis. This effect could be due to the corticoid inhibitory activity on the production of TNF-α [40,41], corroborating previous observations that dexamethasone can prevent necrosis when applied up to 15 minutes after the injection of the *B*. *jararaca* venom in mice [42].

When injected 10 or 45 minutes after the venom, the antivenom alone or in combination with dexamethasone was efficient in reducing the muscle lesions induced by the intramuscular injection of the *B*. *atrox* venom. The creatine kinase level in the group treated with antivenom + dexamethasone was not different from that observed in the animals that were treated only with the antivenom. In this case, the neutralization of toxins by the antivenom seemed to prevent the progression of the muscle lesion and suggested that myotoxins in the *B*. *atrox* venom directly induce this muscle damage independently of the inflammatory response as was described for the venom of *B*. *asper* [43]. However, other studies have shown that the combination of antivenom and dexamethasone could reduce the muscle lesions induced by *B*. *jararaca* or *B*. *jararacussu* venoms, which suggests that an inflammatory response participates in this process [28]. Nonetheless, the inflammatory process is fundamental for appropriate muscle regeneration. The absence of the inflammatory response or the presence of an exacerbated inflammatory process may lead to poor muscle regeneration or a functional decrease in the regenerated tissue due to the replacement of muscle cells by adipose or fibrotic tissue [44,45].

In this context, the use of the combination of antivenom+dexamethasone proved to be the best therapeutic approach among the experimental treatments used in the present study. After the establishment of the muscle injury, from the 7th through the 21st day, only the group treated with the combination of antivenom and dexamethasone showed a significant difference from the untreated control group, which indicated a more effective protection, resulting in a faster recovery of damaged muscle.

Morphologically, all groups demonstrated a time course of regeneration similar to that described for other types of muscle lesions [46], but in the groups treated with dexamethasone, a less intense inflammatory infiltrate was observed. Despite some controversies regarding the use of corticoids in muscle regeneration [47–49], our results suggest that the use of the combination of dexamethasone and the antivenom was beneficial to the regeneration of the muscle tissue. The use of a single dose of dexamethasone in this study could explain the difference from other studies that used anti-inflammatory drugs over a prolonged period, which affected the muscle regeneration [47–49].

Concerning the hemostatic parameters, the use of the combination of dexamethasone and antivenom did not hinder the restoration of the fibrinogen or platelet levels. It has been suggested that the combination of a thrombin inhibitor and dexamethasone could prevent the fibrinogen and platelet depletion in an experimental model of disseminated intravascular coagulation [50]. In the envenomation by *Bothrops* snake bites, the toxins act directly on fibrinogen or through other factors of the blood coagulation cascade including the endogenous formation of thrombin to induce the coagulopathy [51].

In conclusion, our results show that the *Bothrops* antivenom produced in Brazil is useful for the treatment of the experimental envenomation caused by *B*. *atrox* venom, and also suggest that the use of dexamethasone associated to antivenom reduce the time for the recovery of the edema and muscle damage.

Further clinical trials are needed to confirm both, the efficacy of the *Bothrops* antivenom to treat *Bothrops atrox* snake bites from different Amazon regions, and the benefit of the use of dexamethasone associated with the antivenom therapy to treat *Bothrops* snake bites.
